# Application of machine learning techniques to explore the occurrence of macrophage activation syndrome in Still’s disease: results from the GIRRCS AOSD Study Group and the AIDA Network Still’s Disease Registry

**DOI:** 10.3389/fimmu.2026.1811317

**Published:** 2026-04-14

**Authors:** Piero Ruscitti, Francesco Masedu, Antonio Vitale, Valeria Caggiano, Ilenia Di Cola, Fabiola Atzeni, Jessica Sbalchiero, Giuseppe Lopalco, Florenzo Iannone, Maria Morrone, Daniela Iacono, Flavia Riccio, Mariachiara Visconti, Carla Gaggiano, Francesco Caso, Giacomo Emmi, Andrea Hinojosa-Azaola, Jiram Torres-Ruiz, Eduardo Martín-Nares, Giuliana Guggino, Lidia La Barbera, Ezgi D. Batu, Seza Ozen, Petros P. Sfikakis, Luca Navarini, Luisa Costa, Abdurrahman Tufan, Ibrahim Yahya Cakir, Federico Perosa, José Hernández-Rodríguez, Marcella Prete, Lorenzo Dagna, Corrado Campochiaro, Francesco Ursini, Paolo Sfriso, Sara Bindoli, Henrique A. Mayrink Giardini, Lampros Fotis, Katerina Kourtesi, Gaafar Ragab, Mahmoud Ghanema, Moustafa Ali Saad, Haner Direskeneli, Anastasios Karamanakos, Alessandro Conforti, Francesco La Torre, Micol Frassi, Marcello Govoni, Paola Parronchi, Piercarlo Sarzi-Puttini, Maria Cristina Maggio, Serena Bugatti, Ludovico De Stefano, Jacopo Pelizza, Elena Bartoloni, Donato Rigante, Annamaria Iagnocco, Ombretta Viapiana, Ewa Wiesik-Szewczyk, Joanna Makowska, Özgül Soysal Gündüz, Şükran Erten, Benson Ogunjimi, Gian Domenico Sebastiani, Emanuela Del Giudice, Edoardo Biancalana, Giovanni Conti, Ibrahim A. AlMaghlouth, Luciana Breda, Antonio Gidaro, Patrizia Barone, Alma Nunzia Olivieri, Francesco Carubbi, Amato De Paulis, Maria Sole Chimenti, Alberto Lo Gullo, Samar Tharwat, Maissa Thabet, Abdelhfeez Moshrif, Maria Alessio, Angela Mauro, Benoit Suzon, Valentina Pucino, Oksana Boyarchuk, Tetiana Kovalchuk, Cemal Bes, Rabia Deniz, Sulaiman M. Al-Mayouf, Paola Cipriani, Marco Valenti, Alberto Balistreri, Claudia Fabiani, Francesco Ciccia, Carlomaurizio Montecucco, Roberto Giacomelli, Bruno Frediani, Luca Cantarini

**Affiliations:** 1 Department of Biotechnological and Applied Clinical Sciences, University of L’Aquila, L’Aquila, Italy; 2 Department of Medical Sciences, Surgery and Neurosciences, Research Center of Systemic Autoinflammatory Diseases and Behçet’s Disease Clinic University of Siena, Siena, Italy; 3 Azienda Ospedaliero-Universitaria Senese, European Reference Network (ERN) for Rare Immunodeficiency, Autoinflammatory and Autoimmune Diseases (RITA) Center, Siena, Italy; 4 Rheumatology Unit, Department of Experimental and Internal Medicine, University of Messina, Messina, Italy; 5 Department of Precision and Regenerative Medicine and Ionian Area (DiMePRe-J) Policlinic Hospital, University of Bari, Bari, Italy; 6 Department of Precision Medicine, University of Campania “Luigi Vanvitelli”, Naples, Italy; 7 Department of Clinical Medicine and Surgery, University of Naples Federico II, Naples, Italy; 8 Department of Medical, Surgical and Health Sciences, University of Trieste, Trieste, Italy; 9 Clinical Medicine and Rheumatology Unit, Cattinara University Hospital, Trieste, Italy; 10 Centre for Inflammatory Diseases, Department of Medicine, Monash Medical Centre, Monash University, Clayton, VIC, Australia; 11 Department of Immunology and Rheumatology, Instituto Nacional de Ciencias Médicas y Nutrición Salvador Zubirán, Mexico City, Mexico; 12 Rheumatology Section, Department of Health Promotion, Mother and Child Care, Internal Medicine and Medical Specialties, University Hospital P. Giaccone, Palermo, Italy; 13 Department of Pediatric Rheumatology, Faculty of Medicine, Hacettepe University, Ankara, Türkiye; 14 Joint Academic Rheumatology Program, Medical School, National and Kapodistrian University of Athens, Athens, Greece; 15 Clinical and Research Section of Rheumatology and Clinical Immunology, Fondazione Policlinico Campus Bio-Medico, Rome, Italy; 16 Department of Medicine, University of Rome “Campus Biomedico” School of Medicine, Rheumatology, Immunology and Clinical Medicine Unit, Rome, Italy; 17 Gazi University Hospital, Department of Internal Medicine, Division of Rheumatology, Ankara, Türkiye; 18 Department of Interdisciplinary Medicine (DIM), Rheumatic and Systemic Autoimmune Diseases Unit, University of Bari Medical School, Bari, Italy; 19 Department of Autoimmune Diseases, Institut d’Investigacions Biomèdiques August Pi I Sunyer (IDIBAPS), Hospital Clínic of Barcelona [European Reference Network (ERN) for Rare Immunodeficiency, Autoinflammatory and Autoimmune Diseases (RITA) Center], University of Barcelona, Barcelona, Spain; 20 Internal Medicine Unit, Department of Interdisciplinary Medicine, University of Bari Medical School, Bari, Italy; 21 Faculty of Medicine, Università Vita-Salute San Raffaele, Milan, Italy; 22 Unit of Immunology, Rheumatology, Allergy and Rare Diseases, IRCCS Ospedale San Raffaele, Milan, Italy; 23 Medicine & Rheumatology Unit, IRCCS Istituto Ortopedico Rizzoli, Bologna, Italy; 24 Department of Biomedical and Neuromotor Sciences (DIBINEM), Alma Mater Studiorum University of Bologna, Bologna, Italy; 25 Rheumatology Unit, Department of Medicine, University of Padua, [European Reference Network (ERN) for Rare Immunodeficiency, Autoinflammatory and Autoimmune Diseases (RITA) Center], Padua, Italy; 26 Rheumatology Division, Faculdade de Medicina, Hospital das Clínicas, Universidade de São Paulo, São Paulo, Brazil; 27 Department of Pediatrics, Attikon General Hospital, National and Kapodistrian University of Athens, Athens, Greece; 28 Internal Medicine Department, Rheumatology and Clinical Immunology Unit, Faculty of Medicine, Cairo University, Giza, Egypt; 29 Department of Internal Medicine, Division of Rheumatology, Marmara University, Faculty of Medicine, Istanbul, Türkiye; 30 Department of Rheumatology, “Evangelismos” General Hospital, Athens, Greece; 31 U.O. Medicina Generale, Ospedale San Paolo di Civitavecchia, ASL Roma 4, Civitavecchia/Rome, Italy; 32 Department of Pediatrics, Pediatric Rheumatology Center, Giovanni XXIII Pediatric Hospital, University of Bari, Bari, Italy; 33 Rheumatology and Clinical Immunology, Spedali Civili and Department of Clinical and Experimental Sciences, University of Brescia, [European Reference Network (ERN) for Rare Immunodeficiency, Autoinflammatory and Autoimmune Diseases (RITA) Center], Brescia, Italy; 34 Rheumatology Unit, Department of Medical Sciences, Azienda Ospedaliero-Universitaria S. Anna-Ferrara, University of Ferrara, Ferrara, Italy; 35 Department of Experimental and Clinical Medicine, University of Florence, Florence, Italy; 36 Rheumatology Unit, IRCCS Ospedale Galeazzi-S. Ambrogio, Università degli Studi di Milano, Milano, Italy; 37 University Department of Health Promotion, Mother and Child Care, Internal Medicine and Medical Specialties (PROMISE) “G. D’Alessandro”, University of Palermo, Palermo, Italy; 38 Department of Internal Medicine and Therapeutics, University of Pavia, Pavia, Italy; 39 Division of Rheumatology, IRCCS Policlinico San Matteo Foundation, Pavia, Italy; 40 Rheumatology Unit, Department of Medicine and Surgery, University of Perugia, Perugia, Italy; 41 Department of Life Sciences and Public Health, Fondazione Policlinico Universitario A. Gemelli IRCCS, Rome, Italy, Periodic Fever Research Center, Università Cattolica Sacro Cuore, Rome, Italy; 42 Academic Rheumatology Centre, Dipartimento Scienze Cliniche e Biologiche, Università degli Studi di Torino, Torino, Italy; 43 Rheumatology Unit, Department of Medicine, University and Azienda Ospedaliera Universitaria Integrata of Verona, Verona, Italy; 44 Department of Internal Medicine, Pneumonology, Allergology, Clinical Immunology and Rare Diseases, Military Institute of Medicine, National Research Institute, Warsaw, Poland; 45 Department of Rheumatology, Medical University of Lodz, Lodz, Poland; 46 Division of Rheumatology, Department of Internal Medicine, School of Medicine, Manisa Celal Bayar University, Manisa, Türkiye; 47 Department of Rheumatology, Faculty of Medicine Ankara City Hospital, Ankara Yıldırım Beyazıt University, Ankara, Türkiye; 48 Antwerp Center for Translational Immunology and Virology (ACTIV), Center for Health Economics Research and Modeling Infectious Diseases (CHERMID), Vaccine and Infectious Disease Institute (VAXINFECTIO), University of Antwerp, Antwerp, Belgium; 49 Division of Paediatric Rheumatology, Department of Paediatrics, Antwerp University Hospital (UZA), Antwerp, Belgium; 50 Division of Paediatric Rheumatology, Department of Paediatrics, Kidz Health Castle Universitair Ziekenhuis Brussel (UZB), Jette, Belgium; 51 Division of Paediatric Rheumatology, Department of Rheumatology, Ziekenhuis Aan de Stroom (ZAS), Antwerp, Belgium; 52 UOC di Reumatologia, Azienda Ospedaliera San Camillo Forlanini, Roma, Italy; 53 Pediatric and Neonatology Unit, Department of Maternal Infantile and Urological Sciences, Sapienza University of Rome, Latina, Italy; 54 Pediatric Nephrology and Rheumatology Unit, Azienda Ospedaliera Universitaria (AOU), “G. Martino”, Messina, Italy; 55 Rheumatology Unit, Department of Medicine, College of Medicine, King Saud University, Riyadh, Saudi Arabia; 56 College of Medicine Research Center, College of Medicine, King Saud University, Riyadh, Saudi Arabia; 57 Department of Paediatrics, University of Chieti-Pescara, Chieti, Italy; 58 Department of Biomedical and Clinical Sciences Luigi Sacco, Luigi Sacco Hospital, University of Milan, Milan, Italy; 59 Pediatric Rheumatology Unit, Department of Integrated Maternal-Child and Reproduction Activity, AOU “Policlinico-San Marco”, Catania, Italy; 60 Department of Woman, Child and of General and Specialized Surgery, University of Campania “Luigi Vanvitelli”, Naples, Italy; 61 Department of Life, Health and Environmental Sciences and Internal Medicine and Nephrology Unit, Department of Medicine, University of L’Aquila and ASL Avezzano-Sulmona-L’Aquila, San Salvatore Hospital, L’Aquila, Italy; 62 Department of Translational Medical Sciences, Section of Clinical Immunology, University of Naples Federico II, Naples, Italy, Center for Basic and Clinical Immunology Research (CISI), WAO Center of Excellence, University of Naples Federico II, Naples, Italy; 63 Rheumatology, Allergology and Clinical Immunology, Department of Systems Medicine, University of Rome Tor Vergata, Rome, Italy; 64 UOSD REUMATOLOGIA, Ospedale Papardo, Messina, Italy; 65 Rheumatology and Immunology Unit, Internal Medicine Department, Mansoura University, Mansoura, Egypt; 66 Department of Internal Medicine, Faculty of Medicine, Horus University, New Damietta, Egypt; 67 Internal Medicine Department, Farhat Hached University Hospital, University of Sousse, Faculty of medicine of Sousse, Sousse, Tunisia; 68 Rheumatology Department, Faculty of Medicine, Al-Azhar University, Assiut, Egypt; 69 Department of Translational Medical Science, Federico II University of Naples, Naples, Italy; 70 Pediatric Rheumatology Unit, Department of Childhood and Developmental Medicine, Fatebenefratelli-Sacco Hospital, Milan, Italy; 71 EpiCliV Research Unit, University of the French West Indies, Martinique University Hospital, Fort-de France, Martinique; 72 Department of Internal Medicine, Martinique University Hospital, Fort-de France, Martinique; 73 Clinical Immunology and Allergy Unit, Department of Clinical and Experimental Medicine, University of Pisa, Pisa, Italy; 74 Department of Children’s Diseases and Paediatric Surgery, I. Horbachevsky Ternopil National Medical University, Ternopil, Ukraine; 75 Division of Rheumatology, Department of Internal Medicine, Istanbul Basaksehir Cam and Sakura Hospital, Istanbul, Türkiye; 76 Department of Pediatric Rheumatology, King Faisal Specialist Hospital and Research Center, Riyadh, Saudi Arabia; 77 Bioengineering and Biomedical Data Science Lab, Department of Medical Biotechnologies, University of Siena, Siena, Italy; 78 Ophthalmology Unit, Department of Medicine, Surgery and Neurosciences, University of Siena, Siena, Italy

**Keywords:** Still’s disease, systemic juvenile idiopathic arthritis, adult onset Still’s disease, macrophage activation syndrome, machine learning

## Abstract

**Objectives:**

This study aims to explore the application of machine learning techniques in assessing macrophage activation syndrome (MAS) in Still’s disease.

**Methods:**

A multicenter, observational, prospective study was conducted, including patients with Still’s disease enrolled in the Gruppo Italiano di Ricerca in Reumatologia Clinica e Sperimentale (GIRRCS) AOSD Study Group and the AutoInflammatory Disease Alliance (AIDA) Network Still’s Disease Registry.

**Results:**

A total of 737 patients (age: 35.5 ± 17.8, male sex: 44.7%) with Still’s disease were assessed; 11.4% were affected by MAS, and 3% had a poor prognosis. First, random forest imputation was applied to the original dataset. Subsequently, a machine-learning-driven assessment was developed to explore MAS occurrence. Collectively, regression models, an exploration decision tree, and a random forest were applied, suggesting the importance of ferritin, age, C-reactive protein (CRP), and systemic score. A logistic regression model accounting for data leakage concerns was then generated using these variables, and missing values were imputed using random forest imputation. This analysis supported the role of the selected variables, which were further combined across different clinical scenarios to estimate the probability of MAS. The highest risk of MAS was estimated for patients simultaneously characterized by age ≥ 45 years, ferritin ≥ 4,178.10 ng/mL, CRP ≥ 27.15 mg/L, and a systemic score ≥ 7, corresponding to a 34.7% probability of MAS, as well as for those characterized by ferritin ≥ 4,178.10 ng/mL, CRP ≥ 27.15 mg/L, and systemic score ≥ 7, corresponding to a 33.5% probability of MAS.

**Conclusions:**

A machine-learning-driven prediction of MAS was explored in Still’s disease, highlighting the importance of age of onset, hyperferritinaemia, increased CRP, and multiorgan involvement. A combination of these features may suggest a clinician-friendly algorithm for stratifying the probability of MAS during Still’s disease.

## Introduction

Still’s disease is an inflammatory disorder of unknown origin, typically presenting with the clinical symptomatologic triad of high spiking fever, arthritis, and evanescent salmon-colored skin rash associated with a suggestive hyperferritinemia (1, 2). Previously known as systemic juvenile idiopathic arthritis (sJIA) and adult-onset Still’s disease (AOSD), it affects both children and adults along a continuum based on a shared genetic background, pathogenic mechanisms, clinical manifestations, and similar therapeutic strategies (3, 4). From a mechanistic point of view, a unique Still’s disease inflammatory topography has recently been suggested at the crossroads of autoinflammatory and autoimmune disorders (4). The aberrant activation of both innate and adaptive arms of the immune system is recognized as leading to disease occurrence and its clinical manifestations (5). Along with the cardinal manifestations, Still’s disease may present with varying degrees of multiorgan involvement, ranging from mild to severe, depending on different clinical clusters and disease courses (6). Based on this inflammatory clinical picture, patients are treated with glucocorticoids (GCs), conventional synthetic disease-modifying antirheumatic drugs (csDMARDs), and biologic DMARDs (bDMARDs) (7–9). The latest recommendations for the management of Still’s disease suggest prioritizing the use of bDMARDs, primarily interleukin (IL)-1 and IL-6 inhibitors, to increase the rate of patient clinical response and thereby reduce the need for GCs and flares over time (9).

Furthermore, the clinical course of Still’s disease may be complicated by the development of life-threatening complications, mainly macrophage activation syndrome (MAS) (10). This is a secondary form of hemophagocytic lymphohistiocytosis (HLH) that manifests with continuous high fever, hepatosplenomegaly, and marked hyperferritinemia (11). Commonly associated with histological evidence of hemophagocytosis in the bone marrow, the occurrence of MAS delineates a clinical subset of more aggressive Still’s disease characterized by a poor prognosis (10, 11). The challenging clinical scenario of MAS is related to the development of a hyperinflammatory state that rapidly evolves into multiorgan failure syndrome (12, 13). Therefore, timely recognition of MAS is critical for managing this life-threatening progression. In this context, multiple lines of evidence suggest the clinical utility of prognostic tools and laboratory features (10, 14, 15). In addition, some mechanistic biomarkers, which more closely reflect the underlying pathogenic pathways, have also been proposed to improve the accuracy of identifying patients who develop MAS (16, 17). However, the prompt identification of these patients remains crucial, suggesting the need for further improvements in the recognition of MAS and more accurate risk stratification. In this context, the application of advanced machine learning models may offer a novel lens to analyze patients with Still’s disease who develop MAS by simultaneously integrating diverse clinical manifestations (18). These strategies may provide predictions with unprecedented precision, offering insights directly applicable to clinical practice. In fact, these technologies may inform a precision medicine approach, offering clinicians more accurate predictive tools regarding life-threatening evolution and more effective treatment tailoring for the patient’s clinical picture (18–20). However, the application of these technologies in Still’s disease and MAS remains to be fully clarified and established.

On this basis, in this work, we aimed to explore the potential application of machine learning techniques to assess the occurrence of MAS in Still’s disease in a multicenter, observational, prospective study, providing a risk stratification based on the probability of this complication. The machine learning techniques were applied primarily to predict the occurrence of MAS by exploiting a patient’s risk profile and by assessing a pool of variables that can be readily transferred to clinical practice. We also assessed the survival impact of MAS on survival in this study cohort.

## Patients and methods

### Study design, patients, and settings

A multicenter, observational, prospective study was conducted, including patients with Still’s disease enrolled in the Gruppo Italiano di Ricerca in Reumatologia Clinica e Sperimentale (GIRRCS) AOSD Study Group and the AutoInflammatory Disease Alliance (AIDA) Network Still’s Disease Registry. Patients were included before the latest European Alliance of Associations for Rheumatology; PReS: Paediatric Rheumatology European Society (EULAR/PReS) recommendations; thus, children fulfilled specific criteria for sJIA, and for adults, for AOSD, respectively (21–24). In all patients, other inflammatory diseases, malignancies, and infections were ruled out as previously detailed (6, 14). Patients and the public were not involved in the research process.

The GIRRCS AOSD Study Group cohort is a national Italian multicenter cohort involving different rheumatologic centers with extensive experience in the management of Still’s disease as well as in observational studies. Furthermore, patients with Still’s disease were selected from those included in the AIDA Network Still’s Disease Registry, which is an international, clinical, physician-driven, nonpopulation-based, and electronic registry (21). For the Italian centers included in both registries, patients were considered only once to avoid any duplication. Data from patients were prospectively recorded during scheduled visits between January 2020 and December 2024. The main aim of the present work was to explore the application of machine learning techniques in evaluating the occurrence of MAS, leveraging a patient risk profile by assessing a set of variables intended for practical use in clinical practice.

The Ethics Committees of *ASL1 Avezzano-Sulmona-L’Aquila*, L’Aquila, Italy (Ref. No. 0139815/16; 0095184/20) and *Azienda Ospedaliero-Universitaria Senese*, Siena, Italy (Ref. Np. 14951; NCT05200715) approved the study, which was performed in accordance with Good Clinical Practice guidelines and the latest version of the Declaration of Helsinki. Written informed consent was obtained from all participating patients. Clinical data were handled in accordance with the EU General Data Protection Regulations (GDPR) and other applicable regulations governing the processing of personal data and the protection of privacy (2016/679/EU).

The STROBE checklist was followed when reporting the results (**Supplementary Material 1**).

### Clinical variables to be assessed

The clinical features, at the time of diagnosis, were recorded during the scheduled visits. Specifically, we registered fever, typical rash, arthralgia or arthritis, myalgia, lymphadenopathy, sore throat, splenomegaly, hepatomegaly or abnormal liver function tests, and abdominal pain. The diagnosis of pleural effusion or pleuritis and lung parenchymal involvement was made by performing a chest radiograph and computed tomography (CT) scan. Patients with clinical suspicion of pericarditis underwent echocardiography. Combining these features, the systemic score was calculated for each patient as previously described (14, 25). In particular, this score assigns 1 point to each of 12 manifestations: fever, typical rash, pleuritis, lung disease, pericarditis, liver involvement (hepatomegaly or abnormal liver function tests), splenomegaly, lymphadenopathy, leukocytosis > 15,000/mm^3^, sore throat, myalgia, and abdominal pain (maximum score: 12 points). Furthermore, laboratory inflammatory markers, including erythrocyte sedimentation rate (ESR), C-reactive protein (CRP), and ferritin, were recorded at the time of diagnosis.

In addition, at the time of diagnosis and during each scheduled examination, each patient was assessed, where appropriate, for the presence of Still’s disease-related complications, including MAS, lung disease, and others, as suggested by the available literature (10). MAS diagnosis was defined according to the diagnostic criteria proposed in the available literature (26–29). These were the data collected in our study design, taking into account the differences in access to healthcare, considering that the AIDA network is a worldwide registry. The accuracy of these classification criteria is well known and widely used in both research studies and clinical practice. In addition, in our study, the heterogeneity of the population should be considered. In fact, we included patients of all ages according to the proposed Still’s disease continuum. The choice to use different codification criteria was made at the beginning of the study to include patients with MAS and to minimize as much as possible a possible misclassification bias. Lung disease was defined as parenchymal pulmonary involvement due to the disease, as previously reported (30).

Concerning the administration of therapies, GCs, csDMARDs, and bDMARDs were recorded at the time of diagnosis and throughout the subsequent follow-up. Treatment was categorized according to the medications administered to each patient for the longest time period, as previously performed (6, 15, 27, 30). During the available follow-up, based on different disease courses, patients were classified into four groups: one of three clinical patterns (i.e., monocyclic, polycyclic, chronic) or death, depending on the disease course (6, 15, 27, 30). Therefore, only patients with adequate prospective follow-up of at least 12 months were assessed to enable the proper identification of the different disease patterns according to available definitions, unless the patient’s death during follow-up was due to Still’s disease-related causes.

### Data sources, bias, and sample size

During scheduled patient visits, relevant data were collected by reviewing clinical charts stored at each center. All data were fully anonymized before the analyses. All data generated by the analyses are included in the body of the present work. The Research Electronic Data Capture (REDCap) tool was used to collect and store data, as detailed elsewhere (26).

Due to the observational design, this study may have been subject to possible biases, which we attempted to minimize through careful definition of each collected variable. Based on the “real-life” nature of our assessment, no specific sample size was estimated for this study cohort. However, considering the number of patients assessed (*n* = 737), we had sufficient power to perform all of our analyses.

### Statistical analysis

The statistical analysis was conducted using the statistical software R, version 4.4.2. Descriptive statistics were provided for both categorical and continuous variables using frequencies, means, and standard deviation. The study aimed to select and characterize covariates affecting MAS both in terms of the probability of occurrence and survival impact. Both endpoints were subjected to the preliminary issue of missingness. Survival according to MAS was the first assessment of this study. Kaplan–Meier product-limit curves were generated. Differences between Kaplan–Meier survival curves were tested using the log-rank test with a 0.05 level of statistical significance. A multivariate Cox regression model was also performed to estimate hazard rates, particularly those associated with MAS. The proportional hazards assumption was tested using Schoenfeld residuals. The fit of the multivariate model was assessed using the likelihood ratio test.

**Figure 1:**
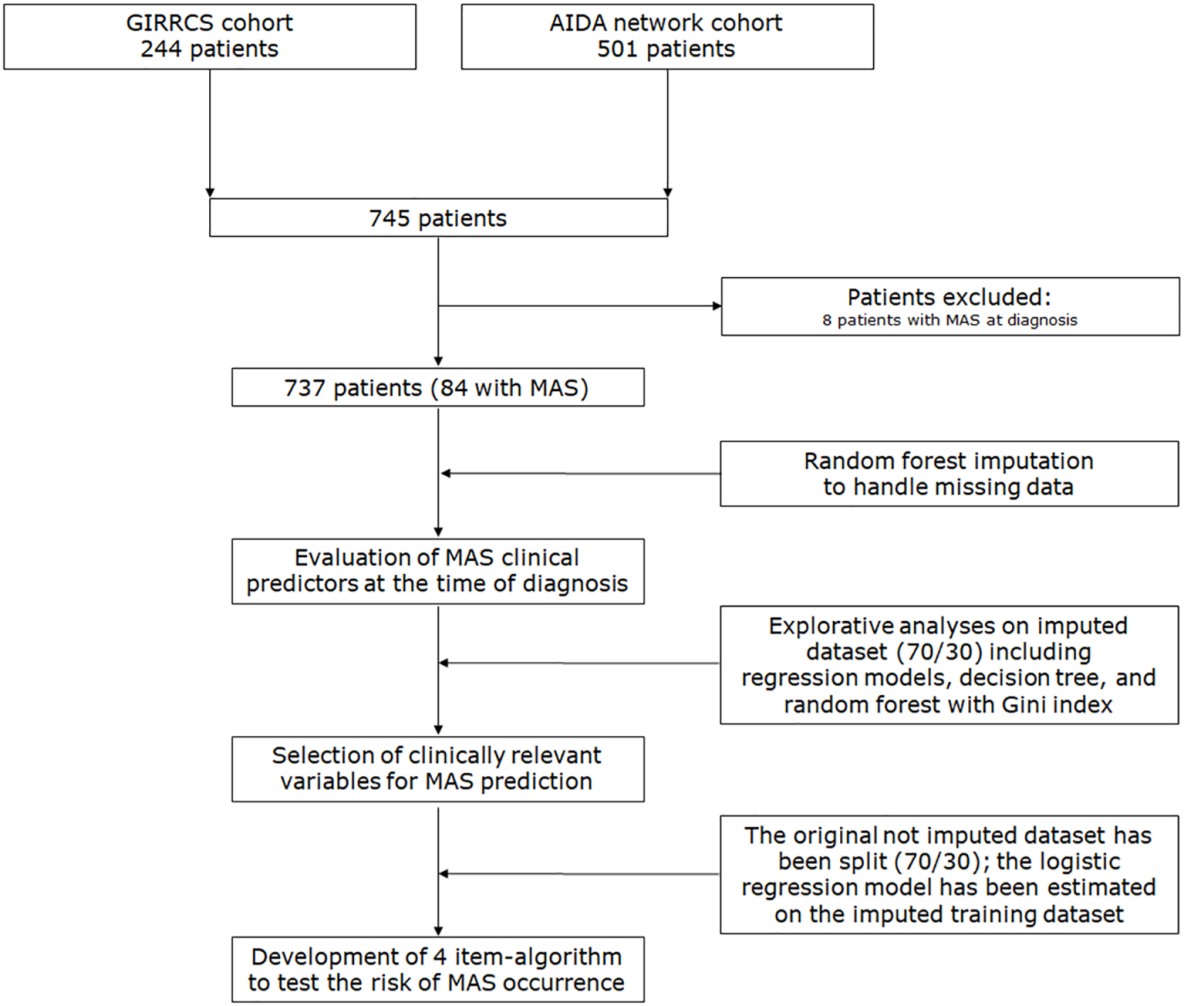
Study flow chart. The study flow chart and the main machine learning methodological steps are presented here.

The main machine learning methodological steps are reported in **Figure 1** and below:

Step 1. Random forest imputation (handling of missing data in the entire dataset): This approach filled in the missing values in the original dataset, capturing nonlinearities and interactions. The outcome (MAS) was excluded from the imputation. As true missing values were unknown, imputation performance was assessed using out-of-bag (OOB) imputation error from missForest. Moreover, distributional were carried out mainly for continuous variables. All these steps were performed prior to modeling.
Step 2. Exploratory assessment without data leakage (train/test split): The imputed dataset was split (70/30) to preliminarily assess the explicative values of the variables for MAS prediction. An exploratory assessment of the outcome was conducted in the postrandom forest-imputed dataset without leakage concerns, using multivariate logistic regression models, decision trees, and random forests. This step provided information on the relevance of variables in predicting MAS.
Step 3. Multivariate logistic regression accounting for data leakage: A 70/30 data split was performed on the original, nonimputed dataset. Random forest imputation was applied to the training set, which provided the dataset for model estimation, and the model was evaluated on the nonimputed test set. The imputation structure learned from the training data was applied to the test set, thereby avoiding potential data leakage.
Step 4. Evaluation of logistic regression performance: Model performance was assessed using specific metrics. To enhance clinical interpretability, the logit score, based on the exploratory analyses, was calculated using the dichotomized selected variables.

### Random forest imputation

In the context of large-scale clinical observational datasets, it is common to encounter missing data that affect many types of statistical analyses requiring complete records. The issue of listwise deletion of records has been address using random forest imputation (31). In our dataset, variables with minimal missingness (< 1%) included sex (one missing); csDMARDs, MTX, bDMARDs, and death (one missing each); MAS (three missing); and age (six missing); these are negligible and unlikely to significantly impact the analyses. Variables with moderate missingness (5%–15%) included joint involvement (89 missing, ~ 12%), fever (70 missing, ~ 9.5%), skin rash (26 missing, ~ 3.5%), pleurisy (33 missing, ~ 4.5%), lung disease (30 missing, ~ 4%), pericarditis (34 missing, ~ 4.6%), liver involvement (30 missing, ~ 4%), splenomegaly (28 missing, ~ 3.8%), lymphadenopathy (29 missing, ~ 3.9%), myalgia (32 missing, ~ 4.3%), abdominal pain (33 missing, ~ 4.5%), sore throat (34 missing, ~ 4.6%), and systemic score 0–12 and systemic score > 7 (74 missing, ~ 10%). These variables might require careful handling, either via imputation or sensitivity analysis. We also had variables with high missingness (> 20%; White Blood Cells (WBC) > 15,000 mm^3^: 252 missing, ~ 34%; ESR: 191 missing, ~ 26%; CRP: 153 missing, ~ 21%, ferritin: 218 missing, ~ 30%; dose of GCs: 224 missing, ~ 30%; follow-up expressed in years: 112 missing, ~ 15%). These variables had substantial missingness and could significantly affect analyses. Imputation or robust missing-data methods (such as random forest imputation) may be appropriate in this context. Thus, these features could justify the use of “random forest imputation”, also considering the worldwide context of the AIDA network and the fact that Still’s disease is a rare condition, making the assembly of large datasets difficult. In fact, beyond statistical considerations, it is crucial to emphasize the value of a robust imputation procedure when working with rare diseases. These conditions are particularly susceptible to bias from missing data due to small sample sizes, where the loss of even a few observations could substantially affect model estimates and overall study conclusions. Given the multivariate distribution to be imputed, and to avoid questionable *a priori* distributional assumptions, we used a nonparametric imputation method capable of handling mixed variable types, accounting for complex interactions, high dimensionality, and nonlinear relations among the variables included in the study design. The only reasonable assumption in our setting is that the observations are pairwise independent. OOB imputation error was estimated at 0.18. Stekhoven and Buhlmann provided reasons supporting this estimate as an appropriate representation of the true imputation error (32). Little’s test for the Missing Completely at Random (MCAR) assumption regarding the multivariate distribution of missingness was preliminarily performed (33). The R libraries “missForest” and “naniar” were used for the imputation and to perform the MCAR hypothesis test, respectively. Random forest-based imputation was chosen because it is a powerful strategy for improving analytical robustness and pattern detection when working with large but sparse datasets, an issue that is particularly relevant in rare-disease research, where assembling large, complete datasets is especially challenging. This approach was adopted to enhance the value of this study in Still’s disease by leveraging a large dataset while mitigating the impact of missingness.

### Clinical predictors’ importance assessment of MAS occurrence

The covariates’ impact on MAS has been assessed by fitting a logit model trained on 70% of the source dataset and tested on the remaining 30%. Variable selection was based on the best-performing Akaike information criteria (AIC) statistics. Variance Inflation Factor (VIF) analysis did not lead to the exclusion of any covariates; the highest value was 1.79 for the systemic score. Odds ratios were derived from the trained multivariate logistic regression model. The confusion matrix yielded a misclassification error of 12.8%. The model showed a high specificity level (99%) with low sensitivity (4%) at an optimal Youden index threshold of 0.52, which could be adjusted according to clinical diagnostic needs.

### Explorative tree analysis and random forest model

Decision tree and random forest models were applied as exploratory tools to assess nonlinear associations and variable importance, rather than as optimized classifiers aimed at maximizing predictive performance.

An exploratory preliminary decision tree, trained on 70% of the original records, was drawn to provide insights into possible clinical decision paths. The tree was estimated on the imputed data using the R library “rpart”, with results using plotted using the “rpart.plot” library. The decision tree was tuned using the complexity parameter (cp = 0.01), the minimum number of observations required to split a node (minsplit = 20), and the minimum depth of the tree (mindepth = 5). The estimated misclassification error was 14.5%. Both the tree and the associated confusion matrix were reported. A random forest model was used to assess the classification importance of the covariates, using 500 trees and four variables tried at each split. The OOB estimate of the error rate was 11.4%. Importance was characterized in terms of the decrease in the Gini index. The results are presented using a random forest variable importance plot.

### Multivariate model with imputed variables in the test set to prevent data leakage

Based on exploratory findings from the regression models, age, ferritin, CRP, and systemic score were considered relevant clinical features for predicting MAS. To improve clinical interpretability, these variables were dichotomized. Missing values for the predictors age, ferritin, CRP, and systemic score were imputed in the training set using random forest imputation. Imputation quality was assessed using OOB error estimates. Subsequently, a logistic regression model was fitted on the imputed training data using only observations with observed outcomes. The imputation structure learned from the training data was then applied to the test set predictors, and model performance was evaluated on the independent test set using discrimination (AUC) and classification accuracy, thereby avoiding data leakage.

The random forest imputation showed heterogeneous performance across variable types. For continuous variables, the OOB error was high (NRMSE: Normalized Root Mean Square Error (NRMSE) = 1.04), indicating poor predictive accuracy of the imputation model for variables such as age, ferritin, CRP, and clinical scores. An NRMSE greater than 1 suggested that the imputed values did not substantially improve over naive marginal predictions, likely reflecting a high proportion of missing data, strongly skewed distributions, and weak correlations among covariates. In contrast, the imputation of categorical variables performed reasonably well, with a proportion of falsely classified (PFC) values of 0.21, consistent with the known ability of random forest imputation to handle binary variables effectively.

## Results

### Descriptive statistics

In this study, 737 patients (age 35.5 ± 17.8, male sex = 44.7%) were assessed, as detailed in **Table 1**, which reports the clinical characteristics of patients before imputation. Of these, 16.7% were pediatric patients. Briefly, all patients had fever, 96.9% showed joint involvement as arthralgia and/or arthritis, and 66.2% experienced skin rash. Evaluating multiorgan involvement, a systemic score of 5.6 ± 1.9 was observed, with 16.1% of patients characterized by a systemic score ≥ 7. A marked increase in CRP (41.2 [37.0] mg/L), ESR (73.9 ± 32.3 mm/h), and ferritin (1,475.0 (4,356) ng/mL) was recorded. Almost all patients received GCs (94.0%) as monotherapy or in association with csDMARDs (67.0%) and/or bDMARDs (48.9%), respectively.

**Table 1:** Descriptive characteristics of assessed patients evaluated in the preimputation dataset analysis.

*Clinical characteristics*	*737 patients*
Demographic features	
Age (years, mean ± SD)	35.5 ± 17.8
Male sex (*n*, %)	329 (44.7)
Disease characteristics	
Fever (*n*, %)	737 (100.0)
Joint involvement^a^ (*n*, %)	628 (96.9)
Skin rash (*n*, %)	471 (66.2)
Sore throat (*n*, %)	397 (56.5)
Myalgia (*n*, %)	403 (57.2)
Lymphadenomegaly (*n*, %)	356 (50.3)
Liver involvement (*n*, %)	301 (42.6)
Splenomegaly (*n*, %)	282 (39.8)
Pleuritis (*n*, %)	109 (15.5)
Pericarditis (*n*, %)	108 (15.3)
Abdominal pain (*n*, %)	95 (13.5)
Systemic score (mean ± SD)	5.6 ± 1.9
Systemic score ≥ 7 (*n*, %)	107 (16.1)
Life-threatening complications	
MAS (*n*, %)	84 (11.4)
Lung disease (*n*, %)	54 (7.6)
Laboratory markers	
CRP (mg/L, median, IQR)	41.2 (37.0)
ESR (mm/h, mean ± SD)	73.9 ± 32.3
Ferritin (ng/mL, median, IQR)	1,475.0 (4,356)
WBC ≥ 15,000 cells/mm^3^ (*n*, %)	326 (67.2)
Therapies	
GCs (*n*, %)	673 (94.0)
Low dosage of GCs (*n*, %)	265 (35.9)
csDMARDs (*n*, %)	493 (67.0)
MTX (*n*, %)	313 (42.5)
bDMARDs (*n*, %)	360 (48.9)
IL-1 inhibitors (*n*, %)	265 (35.9)
IL-6 inhibitor (*n*, %)	59 (8.1)
Disease courses	
Monocyclic pattern (*n*, %)	232 (32.9)
Polycyclic pattern (*n*, %)	233 (33.1)
Chronic pattern (*n*, %)	152 (21.6)
Mortality (*n*, %)	22 (3.0)
Follow-up (years, mean ± SD)	2.8 ± 0.6

a Considered arthralgia and/or arthritis.

SD, standard deviation; IQR, interquartile range; CRP, C-reactive protein; ESR, erythrocyte sedimentation rate; IQR, interquartile range; GCs, glucocorticoids; csDMARDs, conventional synthetic disease modifying antirheumatic drugs; bDMARDs, biologic disease-modifying antirheumatic drugs.

In this cohort, 11.4% of patients were burdened by MAS, and 3% had a poor prognosis; the presence of MAS was estimated to be correlated with mortality (*χ*^2^ = 33.3, *p* < 0.001).

Specifically, patients died due to multiorgan dysfunction associated with uncontrollable MAS. The development of MAS occurred between 1 and 12 months (median, 6 months) after the diagnosis of Still’s disease in this cohort of patients. According to the analysis, 100% of patients fulfilled the 2016 EULAR/ACR criteria sensitivity, whereas 83% met the HLH-2004 criteria (28, 29). In addition, the occurrence of MAS correlated with ferritin (*r* = 0.12, *p* = 0.008) and with the systemic score (*r* = 0.23, *p* < 0.001), whereas it was not associated with either CRP (*r* = 0.05, *p* = 0.180) or ESR (*r* = 0.01, *p* = 0.883).

### Survival analysis

**Figure 2:**
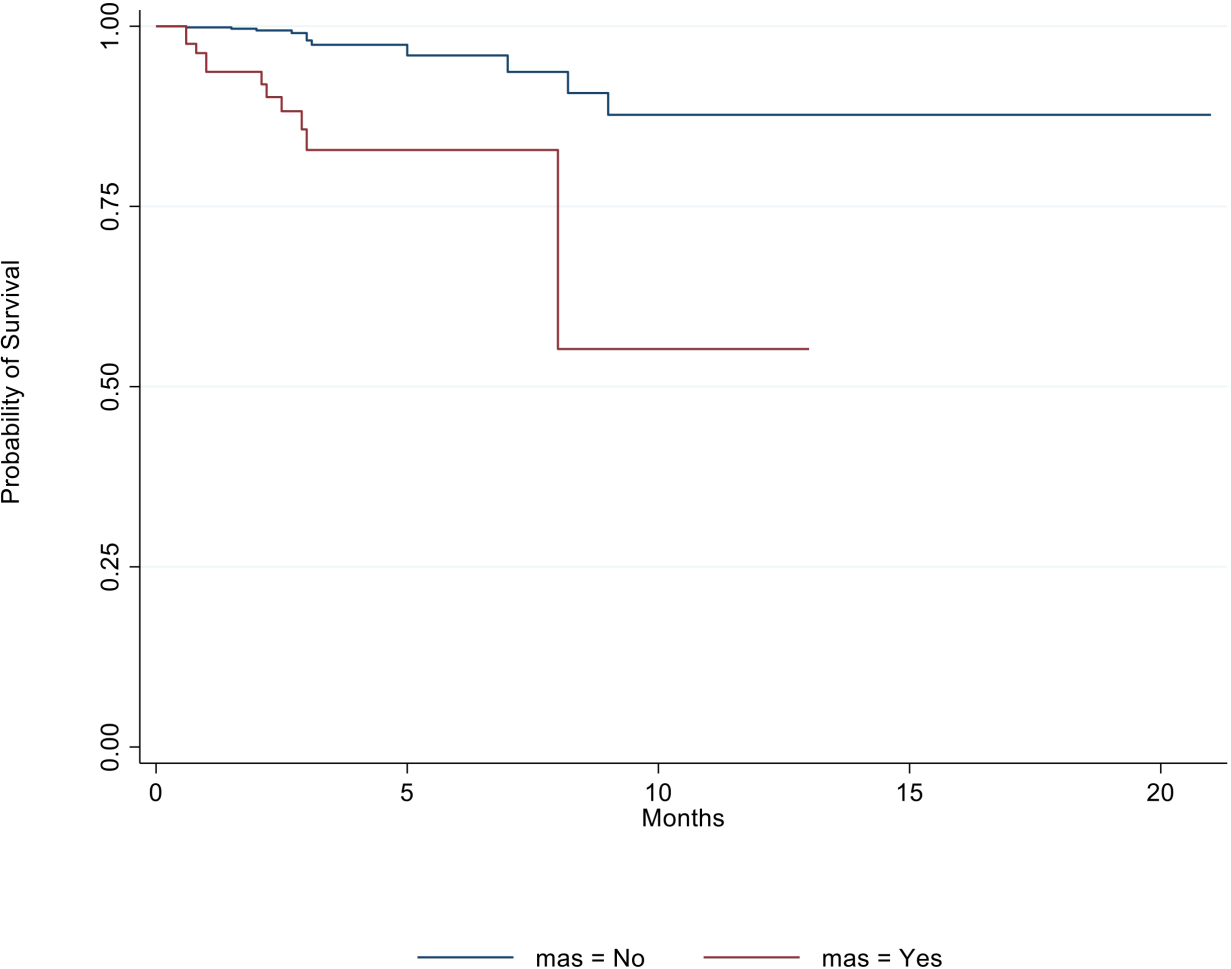
The occurrence of MAS is associated with poor prognosis. Here, the overall survival analysis is presented, stratifying patients based on the presence or absence of MAS. According to the log-rank test, there is a statistically significant difference between the survival of patients with MAS (light blue line) and those without this complication (red line) (*χ*^2^ = 33.8; *p* < 0.001).

The overall survival analysis showed, according to the log-rank test, a statistically significant difference in survival between patients with MAS and those without (*χ*^2^ = 33.8; *p* < 0.001). Given that median survival times were not estimable, the restricted mean survival time (RMST) was calculated (RMSTMAS = No = 19.2, SEMAS = No = 0.65; RMSTMAS = Yes = 14.1, SEMAS = Yes = 3.03), which, together with inspection of the table of patients at risk, confirmed higher mortality in the MAS group, as reported in **Figure 2**. Furthermore, a Cox regression model was performed, including clinical variables with well-known prognostic impact. In this analysis, the presence of MAS (hazard ratio [HR] = 11.29, 95% confidence interval [CI] =4.49–28.41, *p* < 0.001) was identified as the main factor associated with mortality, consistent with a proportional hazards (PH) model. The latter was adjusted for age, sex, CRP, ferritin, and lung disease. Age (HR = 1.03, 95% CI = 1.01–1.05, *p* = 0.005), male sex (HR = 2.94, 95% CI = 1.12–7.72, *p* = 0.028), and lung disease (HR = 2.98, 95% CI = 1.01–8.86, *p* = 0.048) were significant predictors of mortality. The Shoenfeld residuals test (*χ*^2^_global = 5.53; *p* = 0.477) supported the PH assumption. We also performed an additional Cox regression model assessing the role of multiorgan involvement and therapies on mortality. These findings are reported in **Table 2**.

**Table 2:** HR Cox model estimation evaluating predictors of mortality in assessed patients with Still’s disease.

Mortality	HR	95% CI	*p*-value
Multivariate model with life-threatening complications and laboratory markers			
Age	1.03	1.01–1.05	**0.005**
Male sex	2.94	1.12–7.72	**0.028**
MAS	11.29	4.49–28.41	**< 0.001**
Lung disease	2.98	1.01–8.86	**0.048**
CRP	1.00	1.00–1.01	0.636
Ferritin	1.00	1.00–1.02	0.225
Multivariate model with multiorgan involvement			
Age	1.04	1.02–1.06	**< 0.001**
Male sex	2.28	0.88–5.88	0.088
Liver involvement	0.76	0.31–1.89	0.561
Splenomegaly	1.72	0.66–4.44	0.265
Lymphadenomegaly	1.44	0.56–3.69	0.444
Sore throat	1.69	0.67–4.25	0.266
Multivariate model with therapies			
Age	1.04	1.01–1.06	**< 0.001**
Male sex	2.4	0.96–6.03	0.061
Systemic score ≥ 7	3.17	1.31–7.62	**0.010**
csDMARDs	1.38	0.49–3.91	0.541
IL-1 inhibitors	1.08	0.40–2.92	0.874
IL-6 inhibitors	1.91	0.52–7.03	0.327

HR, hazard ratio; 95% CI, 95% confidence interval; MAS, macrophage activation syndrome; CRP, C-reactive protein; csDMARDs, conventional synthetic disease-modifying antirheumatic drugs.Bold values are statistically significant.

Cox regression provided information about the clinical impact of MAS on mortality, forming the basis for subsequent analysis to better codify the risk of MAS.

### Exploratory assessment of the importance of MAS predictors, logistic regression models, decision trees, and random forests for the codification of MAS

Based on the previous findings, MAS remained the main factor influencing the survival of patients with Still’s disease. Therefore, we explored a possible machine-learning-driven assessment to improve the codification of patients developing this complication, potentially refining the evaluation of clinical predictors. Given the explorative nature of such assessment, these analyses were performed on the dataset after random forest imputation, without data leakage concerns. This methodological choice was also made to be as comprehensive as possible in order to maximize the information derived from the collected results.

The logistic regression model was trained using a 70/30 split of the patients’ records, as reported in **Table 3**. The variable selection strategy was based on both clinical and statistical criteria. VIF analysis was performed, and no evidence of multicollinearity was observed among the covariates in this specific model (VIF = 1.14). A systemic score ≥ 7 was a strong predictor of the occurrence of MAS in our cohort of patients with Still’s disease (OR = 2.70, 95% CI = 1.30–5.60, *p* = 0.007). Furthermore, liver involvement (OR = 1.99, 95% CI = 1.01–3.92, *p* = 0.045) and ferritin levels (OR = 1.02, 95% CI = 1.01–1.03, *p* = 0.024) were also predictors of MAS occurrence. Model performance was assessed, with a misclassification rate of 13.3% on the test set. An AIC of 333.92 and an AUC of 0.622 were estimated for this model. Predictor importance was first assessed by training a logistic model to provide OR statistics and evaluating misclassification performance on the test set.

**Table 3:** OR logistic regression model estimation evaluating predictors of MAS in assessed patients with Still’s disease.

MAS	OR	95% CI	*p*-value
Age	0.99	0.97–1.01	0.206
Male sex	0.62	0.34–1.15	0.131
Systemic score ≥ 7	2.70	1.30–5.60	**0.007**
Lung disease	1.54	0.58–4.12	0.386
Lymphadenomegaly	1.92	0.99–3.72	0.053
Liver involvement	1.99	1.01–3.92	**0.045**
Splenomegaly	0.99	0.99–1.91	0.982
CRP	1.00	1.00–1.01	0.597
Ferritin	1.02	1.01–1.03	**0.024**

OR, odds ratio; 95% CI, 95% confidence interval; MAS, macrophage activation syndrome; CRP, C-reactive protein.Bold values are statistically significant.

Decision tree and random forest models were applied as exploratory tools to assess nonlinear associations and variable importance, rather than as optimized classifiers aimed at maximizing predictive performance.

The predictive role played by the clinical features has been preliminarily addressed by training a decision tree, which, according to the confusion matrix, had a misclassification error of 17.3%. The use of machine learning algorithms may suffer from a lack of interpretability, but they may provide important insights into the classification strength of clinical features. This analysis highlighted the importance of a systemic score ≥ 7 in identifying patients with MAS as the main node in the decision tree. Subsequently, the roles of ferritin and age were identified as additional important nodes. Consequently, the relevance of age, CRP, and ESR was also suggested. In addition, different thresholds for the continuous variables were identified, suggesting varying importance of laboratory markers and age according to the patient’s clinical scenario. These findings are reported in **Supplementary Figure 1**.

After the assessment of the explorative decision tree, a random forest was also trained on the training set previously used in logistic regression. This analysis was paired with the training of a random forest model, reporting variable importance using the magnitude of the Gini index. The random forest classification performance was measured using the OOB (11.1%). The random forest confirmed the relevance of ferritin, age, CRP, ESR, and systemic score. This analysis also showed the importance of other clinical features in characterizing patients with MAS. Their mean decrease in the GINI index is reported in **Supplementary Figure 2**.

Using multiple models allowed us to identify features that were consistently relevant across different methods. Logistic regression was chosen as the primary model due to its clear clinical interpretability. The logistic regression analysis employed a training/test split to control for overfitting and focused on the logistic model.

In our study, we prioritized addressing substantial missing data using random forest imputation. While future work could explore rebalancing techniques or threshold adjustments to improve detection of the minority MAS class (~ 11%), we pragmatically chose not to apply rebalancing here, as it could have introduced a two-step intervention—imputation followed by data weighting—and we focused primarily on handling missing values.

### Clinical risk probability of MAS occurrence

Based on the findings of the explorative analyses derived from the above-mentioned logistic regression models, decision tree, and random forest for the codification of MAS, the variables age, ferritin, CRP, ESR, and systemic score were identified as relevant predictors in our cohort. The machine learning procedures enabled us to get a better understanding of the relevance of these variables. These clinical variables were dichotomized using ROC curves to identify the empirically optimal cut-off values for the prediction of MAS. This choice was made to further improve the clinical interpretability of these variables. ESR was excluded from this analysis to avoid redundancy with CRP, whereas the cut-off of ≥ 7 was used for the systemic score according to previous literature (15). The cut-off of 45.0 years was derived for age (AUC: 0.4131; 95% CI = 0.38–0.45; sensitivity: 0.27, specificity: 0.73), 4,178.10 ng/mL for ferritin (AUC: 0.66; 95% CI = 0.63–0.70; sensitivity: 0.57, specificity: 0.68), and 27.15 mg/L for CRP (AUC: 0.55; 95% CI = 0.51–0.59; sensitivity: 0.48, specificity: 0.62). According to these results, a multivariate logistic model was built using these dichotomic variables, as reported in **Table 4**. In this model, ferritin ≥ 4,178.10 ng/mL (OR = 2.58, 95% CI = 1.59–4.17, *p* < 0.001) and systemic score ≥ 7 (OR = 3.84, 95% CI = 2.28–6.46, *p* < 0.001) resulted in significant predictors of the presence of MAS. An AIC of 333.24 was estimated for this model. We also performed this analysis excluding pediatric patients (**Table 4**).

**Table 4:** OR logistic regression model estimation evaluating dichotomized clinical predictors of MAS in assessed patients with Still’s disease.

MAS	OR	95% CI	*p*-value
Multivariate model comprising the pediatric and adult patients			
Age ≥ 45.0 years	0.79	0.46–1.35	0.383
Ferritin ≥ 4,178.10 ng/mL	2.58	1.59–4.17	**< 0.001**
CRP ≥ 27.15 mg/L	1.54	0.96–2.49	0.074
Systemic score ≥ 7	3.84	2.28–6.46	**< 0.001**
Multivariate model comprising only adult patients			
Age ≥ 45.0 years	0.18	0.04–3.05	0.561
Ferritin ≥ 4,178.10 ng/mL	1.77	1.19–2.36	**0.009**
CRP ≥ 27.15 mg/L	1.96	1.37–2.54	**0.001**
Systemic score ≥ 7	1.87	1.28–2.47	**< 0.001**
Multivariate model comprising imputed variables to prevent data leakage			
Age ≥ 45.0 years	0.88	0.47–1.64	0.689
Ferritin ≥ 4,178.10 ng/mL	1.65	0.93–2.95	0.087
CRP ≥ 27.15 mg/L	0.89	0.50–1.60	0.713
Systemic score ≥ 7	3.93	2.16–7.15	**< 0.001**

OR, odds ratio; 95% CI, 95% confidence interval; MAS, macrophage activation syndrome; CRP, C-reactive protein.Bold values are statistically significant.

After that, a logistic model accounting for data leakage concerns was generated, considering age, ferritin, CRP, and systemic score as relevant clinical features in predicting MAS. A 70/30 data split was performed on the original, nonimputed dataset. Random forest imputation was applied to the training set, which provided the dataset used to estimate the model to be evaluated on the nonimputed test set. The imputation structure learned from the training data was then applied to the test set, thereby avoiding potential data leakage. The developed model further suggested the relevance of the selected clinical variables in predicting MAS (AUC = 0.666, AIC = 350.29). An AUC of 0.666 may indicate weak to moderate discriminative performance; however, this aligns with the hypothesis-generating nature of our study. Nevertheless, this finding should be interpreted in light of the exploratory and hypothesis-generating nature of the present study and the complexity of real-world datasets in rare diseases.

Specifically, systemic score ≥ 7 (OR = 3.93, 95% CI = 2.16–7.15, *p* < 0.001) was a significant predictor of MAS, whereas a trend was observed for ferritin ≥ 4,178.10 ng/mL (OR = 1.65, 95% CI = 0.93–2.95, *p* = 0.087). These results are reported in **Table 4** (Multivariate model comprising imputed variables to prevent data leakage). The relative metrics of this specific model are reported in **Supplementary Results 1**.

Finally, combining all these variables across different patient clinical scenarios, the probability of developing MAS was estimated accordingly. The highest risk of MAS was estimated for patients simultaneously characterized by age ≥ 45 years, ferritin ≥ 4,178.10 ng/mL, CRP ≥ 27.15 mg/L, and a systemic score ≥ 7, corresponding to a 34.7% probability of MAS, as well as for those characterized by ferritin ≥ 4,178.10 ng/mL, CRP ≥ 27.15 mg/L, and a systemic score ≥ 7, corresponding to a 33.5% probability of MAS. As reported in **Table 5**, based on the different possible combinations of these clinical characteristics, low-risk and intermediate-risk patients were also identified according to the estimated probabilities of MAS. In addition, our four-item probability algorithm showed a sensitivity of 26.0% and specificity of 94.0%.

**Table 5:** Probability of MAS according to selected clinical variables and different possible patient scenarios in Still’s disease.

Age ≥ 45.0 years	Ferritin ≥ 4,178.1 ng/mL	CRP ≥ 27.2 mg/L	Systemic score ≥ 7	Probability of MAS	Risk categories
−	−	−	−	6.0% (4.5–11.3)	Low risk
+	−	−	−	7.0% (2.5–11.5)	
−	−	+	−	7.1% (3.3–11.6)	
+	−	+	−	7.3% (1.8–18.9)	
+	+	+	−	10.1% (3.8–16.4)	
+	+	+	−	10.8% (3.8–16.4)	
+	+	−	−	11.1% (4.7–17.6)	
−	+	+	−	11.3% (5.0–17.6)	
−	+	−	−	12.5% (6.1–19.8)	
+	−	−	+	24.4% (9.1–36.8)	Intermediate risk
−	−	−	+	25.3% (13.5–37.1)	
−	−	+	+	25.7% (10.4–36.2)	
+	−	+	+	31.1% (6.9–39.3)	High risk
−	+	−	+	32.0% (20.9–42.9)	
+	+	−	+	33.1% (17.7–48.8)	
−	+	+	+	33.5% (18.0–49.1)	
+	+	+	+	34.7% (15.2–49.8)	

CRP, C-reactive protein; MAS, macrophage activation syndrome.

All these analyses were performed and designed to increase the generalizability of the results. In fact, we exploited the probability of MAS according to different combinations of selected variables to ensure proper generalization of the results in clinical practice. Regarding model calibration, metrics such as the Brier score could be used to evaluate the agreement between predicted probabilities and observed outcomes and may provide additional insight into model performance. To address this issue, several methodological precautions were implemented. Model development and evaluation were performed using a train/test split (70/30) conducted on the original nonimputed dataset. Random forest imputation was applied only to the training set, and the learned imputation structure was subsequently applied to the test predictors, allowing the model to be evaluated on independent data and preventing data leakage. This approach represents one of the most widely used strategies to limit overfitting in predictive modeling. Therefore, while the Brier score may provide complementary information on calibration, it would not substantially modify the interpretation of model robustness in our specific analytical framework, where the primary safeguards against overfitting were the use of an independent test set and the adoption of a parsimonious model structure.

## Discussion

In this study, a machine learning-based risk prediction of MAS was explored to potentially increase the accuracy of detecting this condition, which, despite the recent management improvements, remains a life-threatening complication in patients with Still’s disease. Furthermore, age ≥ 45 years, ferritin ≥ 4,178.10 ng/mL, CRP ≥ 27.15 mg/L, and systemic score ≥ 7 were identified as relevant clinical characteristics for risk prediction of MAS, and their combinations may delineate patient subsets with differing risks of this complication in the context of Still’s disease.

In the present multicenter, observational, prospective study, 11.4% of patients were affected by MAS, and 3% of those had a poor prognosis. Despite the observed improvement and the development of specific recommendations for management (34, 35), the presence of MAS is reported to be associated with mortality, influencing the prognosis of Still’s disease (11, 14, 36). Therefore, accurate prediction of this complication is considered crucial for the management of such patients. The significance of machine learning applications in medicine is increasingly highlighted in improving the detection of specific manifestations through the application of these cutting-edge technologies (18–20). In fact, these machine-learning techniques have been gaining popularity as a more comprehensive, “nonlinear”, and accurate method to predict patient clinical scenarios. In particular, a vast number of variables may be simultaneously analyzed to identify combinations that reliably predict diverse outcomes; this approach is considered superior to the classic “linear” predictive tools currently used by clinicians for patient prognostication (18–20).

Furthermore, age ≥ 45 years, ferritin ≥ 4,178.10 ng/mL, CRP ≥ 27.15 mg/L, and systemic score ≥ 7 have been identified as relevant clinical characteristics in the prediction of MAS, and their combinations may delineate some patient subsets with differing risks of this complication in the context of Still’s disease. The probability of MAS presence was also estimated to provide a clinician-friendly algorithm combining these clinical features. In the analyses, after explorative evaluations, a logistic model accounting for data leakage concerns was generated, considering age, ferritin, CRP, and systemic score as relevant clinical features for predicting MAS. A data split (70/30) was performed on the original, nonimputed dataset. Random forest imputation was applied to the training set, which provided the dataset used to estimate the model evaluated on the nonimputed test set. The imputation structure learned from the training data was applied to the test set, avoiding potential data leakage. In addition, an AUC of 0.666 for a clinician-friendly algorithm may indicate weak to moderate discriminative analysis; however, this finding aligns with the hypothesis-generating nature of the study. It should be interpreted in light of the exploratory design of the present study and the complexity of real-world datasets in rare diseases. In such contexts, moderate AUC values are not uncommon and may still provide clinically meaningful signals that warrant further investigation. In fact, the primary aim of the analysis was not to develop a fully optimized predictive model but rather to explore potential clinical predictors of MAS and translate these findings into a clinically interpretable framework. For this reason, the model was intentionally kept parsimonious and based on a limited number of clinically meaningful variables (age, ferritin, CRP, and systemic score) identified across multiple exploratory approaches. Furthermore, the developed four-item probability algorithm showed a sensitivity of 26.0% and a specificity of 94.0%. The sensitivity is lower than that of the HLH-2004 classification criteria, which had a sensitivity 99.0% and a specificity 97.1%, and the 2016 EULAR/ACR classification criteria, which had a sensitivity of 73.0% and a specificity of 99%, respectively (28, 29). The lower sensitivity of the four-item probability algorithm is related to the fact that it provides a “risk-stratification” approach for MAS in Still’s disease; it was designed to produce additional classification criteria. In our study, age ≥ 45 years was associated with MAS. This result may be considered partially conflicting with the available literature, which has increasingly suggested a higher rate of MAS in pediatric patients less than 2 years (37, 38). However, larger studies in adults are still lacking, also considering the reduced disease frequency and poor prognosis when MAS develops, with an estimated mortality of up to 40% (11, 14, 36). Therefore, although the Still’s disease continuum is a concept increasingly reported and accepted by multiple lines of evidence (3, 39), the age of onset may influence the different disease manifestations and consequent patient outcomes. Aging is associated with chronic inflammation, which leads to cellular senescence and organ dysfunction (40). Factors secreted by aged cells and chronic inflammation may further accelerate the senescence of immune cells. This could result in weakened immune function and an inability to clear senescent cells and inflammatory factors, consequently favoring the development of organ damage and aging-related diseases (40). Taken together, these observations suggest a more pronounced vulnerability of adult patients with Still’s disease toward MAS, although further comparative studies across different ages of onset are needed to fully clarify these issues.

In addition, in our cohort, ferritin ≥ 4,178.10 ng/mL was associated with the occurrence of MAS. Although different thresholds have been reported in the available literature (4, 10, 13, 15, 29, 41), a higher ferritin cut-off was derived in our study. This may be related to the presence of patients with particularly high ferritin levels, the so-called hyperferritinemic cluster (6), but it may also reflect differences in study settings and methods. Another possibility is that ferritin is a highly skewed variable, and extreme values may have influenced this estimate. This likely reflects the distribution of ferritin values in the dataset rather than true model overfitting. In this context, the assessment of ferritin may provide useful information that is readily transferable into clinical practice, helping to alert physicians to the possibility of a higher risk of this complication in patients with Still’s disease. In this context, hyperferritinemia may play an important role in differentiating MAS from other forms of HLH (29, 41). Furthermore, ferritin expression in the bone marrow of patients with Still’s disease and MAS has been shown to correlate with peripheral blood cytopenia and the severity of the clinical picture (42). In addition, both Still’s disease and MAS have been included under the so-called hyperferritinemic syndrome, in which the proinflammatory properties of ferritin may contribute to pathogenesis and inflammatory burden (43). Moreover, CRP ≥ 27.15 mg/L was associated with MAS in our cohort of patients. As observed in different disease contexts, CRP levels correlate with organ failure and poor prognosis in patients admitted to intensive care units (44, 45). Following its production, the subsequent release of proinflammatory cytokines may contribute to the development of an aberrant inflammatory process (46), thus possibly linking elevated CRP levels with the evolution of Still’s disease toward MAS. Furthermore, the data also highlighted a systemic score ≥  7 as a predictive factor of the life-threatening evolution of Still’s disease. This systemic score threshold may be readily applied in clinical practice to identify patients with a higher likelihood of a more severe clinical course and poor outcome (15, 27). In our study, patients were assessed at the time of diagnosis, and it remains to be fully established whether the systemic score may increase in proximity to MAS development. Additionally, specifically designed studies are needed to clarify whether the systemic score reflects the severity of clinical presentations related to MAS. Taken together, these observations reinforce the importance of accurate clinical evaluation and prognostication of the patient’s clinical picture, while simultaneously integrating diverse manifestations according to the machine-learning-driven results. In fact, the relevance of age of onset, hyperferritinemia, increased CRP, and multiorgan involvement of the disease may be suggested, as they are directly related to the development of a severe hyperinflammatory state, acute-phase physiological changes, and aberrant immune cell-mediated responses culminating in cytokine-induced tissue damage and the occurrence of MAS in Still’s disease (11–13).

All things considered, the detection of MAS in Still’s disease has been increasingly emphasized and investigated to better stratify the risk of this complication, considering its prognostic impact (45–48). In these studies, different features, including splenomegaly, liver involvement, pericarditis, and neurological symptoms, together with hyperferritinemia, have been variably associated with the occurrence of MAS (46–49). In contrast, the clinician-friendly algorithm combining these clinical features may improve the accuracy of risk prediction for MAS using machine learning techniques. Different ferritin thresholds have been proposed, likely reflecting differences in study designs and settings. Future specific studies are needed to compare the accuracy of different scoring systems in predicting the risk of MAS.

In regression predictive models, we did not incorporate some laboratory markers that are established indicators of MAS in the context of Still’s disease, including WBCs and platelets. Patients with leukopenia and thrombocytopenia may already have developed MAS (50); however, we assessed patients before the occurrence of such a complication to stratify patient risk profiles. Furthermore, our codification of MAS relied on classification criteria based on these laboratory markers (29), thereby limiting the validity of predictive models that include these features. In addition, in our global setting, the availability of assays for assessing NK activity and sCD25, which are included in the classification criteria for HLH (28), was limited. These are well-known markers linked to the occurrence of HLH, but they are not routinely available, limiting their application in daily clinical practice. Furthermore, a growing body of evidence also suggests the role of mechanistic biomarkers, reflecting the involved pathogenic pathways, in predicting MAS during Still’s disease (51–53). In fact, a cytokine profile could better evaluate the full pathophysiological spectrum of MAS in Still’s disease, as shown in previous experience (54); however, this approach may not be feasible in a global setting characterized by varying possibilities to access healthcare resources.

Our study has several limitations that may reduce the generalizability of the derived results. Although we assessed a combination of two large prospective cohorts including patients with Still’s disease, multicenter studies have inherent limitations related to differences in clinical practice between centers, which may affect data collection and, consequently, the interpretation of the results. Furthermore, although Still’s disease is more prevalent in pediatric populations, the study predominantly included adults, supporting the need for further studies in children. Moreover, different therapeutic strategies may have been applied in the management of patients with Still’s disease. Due to the observational nature of the study, therapies were not systematically administered, which may have affected the assessment of their influence on MAS. This may influence the study outcome, highlighting the need for specifically designed and adequately powered studies to address these issues and to assess the impact of therapies. In addition, the AIDA Still’s disease registry is a worldwide international study that may reflect differences in access to healthcare resources, thereby further limiting the possibility of performing specific analyses on administered drugs and the presence of MAS. Our study was not primarily designed to compare our criteria with the H-score. Specifically, the H-score was retrospectively derived in clinical settings different from those of Still’s disease. As such, we did not collect certain items, such as hemophagocytosis and fever degree, required to calculate the H-score, thereby limiting the possibility of performing a direct comparison. Similarly, we did not perform a specific comparison between our derived four-item algorithm and the available classification criteria for MAS and HLH. These comparisons were outside the main scope of the present work, which was primarily devoted to stratifying the risk of MAS occurrence, and some items from the classification criteria were not fully available in our global setting, limiting these possible evaluations. Future, specifically designed studies could address this issue by validating our four-item algorithm. In addition, we did not specifically investigate certain laboratory features and imaging findings that have recently been assessed in another study from the AIDA Network Still’s Disease Registry and previous experiences (55–57). Finally, we intentionally did not apply rebalancing techniques (e.g., SMOTE or class weighting) because the dataset already required substantial imputation. In our opinion, applying both imputation and rebalancing could introduce compounded modeling artifacts, as our primary objective was clinical interpretability and risk stratification rather than maximizing sensitivity in a deployment-ready classifier. Future work specifically using imbalance-aware strategies is needed to fully clarify these issues. Taking together, these observations indicate that the hypothesis-generating nature of our study should be acknowledged, as it explored the potential application of machine learning techniques in predicting MAS in Still’s disease.

In conclusion, this work explored a machine-learning-driven prediction of MAS occurrence in Still’s disease in a multicenter, observational, prospective study. The analyses highlighted the importance of age of onset, hyperferritinemia, increased CRP, and multiorgan involvement. A combination of these features may suggest a clinician-friendly algorithm for identifying the probability of MAS occurrence during Still’s disease. Further studies are needed to fill the gap between promising and comprehensive research on the potential utility of machine learning in the field of Still’s disease and its actual implementation in daily clinical practice to improve patient management. External validation is needed to confirm these findings. The hypothesis-generating nature of the study should be recognized in preliminary studies, providing the basis for further confirmatory studies to improve the management of patients with Still’s disease at high risk of developing MAS.

## Data Availability

The original contributions presented in the study are included in the article/**Supplementary Material**. Further inquiries can be directed to the corresponding authors.

## Supplementary material

Supplementary Figure 1 An explorative decision tree for the occurrence of MAS. In this figure, the diagnostic role played by patient clinical features has been preliminarily addressed through training a decision tree which, according to the confusion matrix, showed a misclassification error of 17.3%. This assessment suggested the importance of systemic score ≥ 7 in identifying patients with MAS as main node in the decision tree. After that, the role of ferritin and age was highlighted as other important nodes. Consequently, the relevance of age, CRP, and ESR was suggested. In addition, different thresholds for the continuous variables were suggested, thus proposing the diverse significance of the laboratory markers and age according to the clinical scenario.

Supplementary Figure 2 Random forest classification performance and Gini’ mean decrease of clinical variables in assessing the risk for the occurrence of MAS. A random forest has been trained on the training set; the random forest classification performance was measured using the OOB (11.1%). The random forest confirmed the relevance of ferritin, age, CRP, ESR, and systemic score. This analysis has also shown the importance of other clinical features in suggesting a more accurate characterization of patients with MAS. The Gini’s mean decrease of assessed clinical variables is also reported in this Figure.
