# Supplementary figures and images for: Application of machine learning techniques to explore the occurrence of macrophage activation syndrome in Still’s disease: results from the GIRRCS AOSD Study Group and the AIDA Network Still’s Disease Registry
Source: Front Immunol. 2026 Apr 14;17:1811317. doi: 10.3389/fimmu.2026.1811317 (PMC13121312; doi:10.3389/fimmu.2026.1811317)

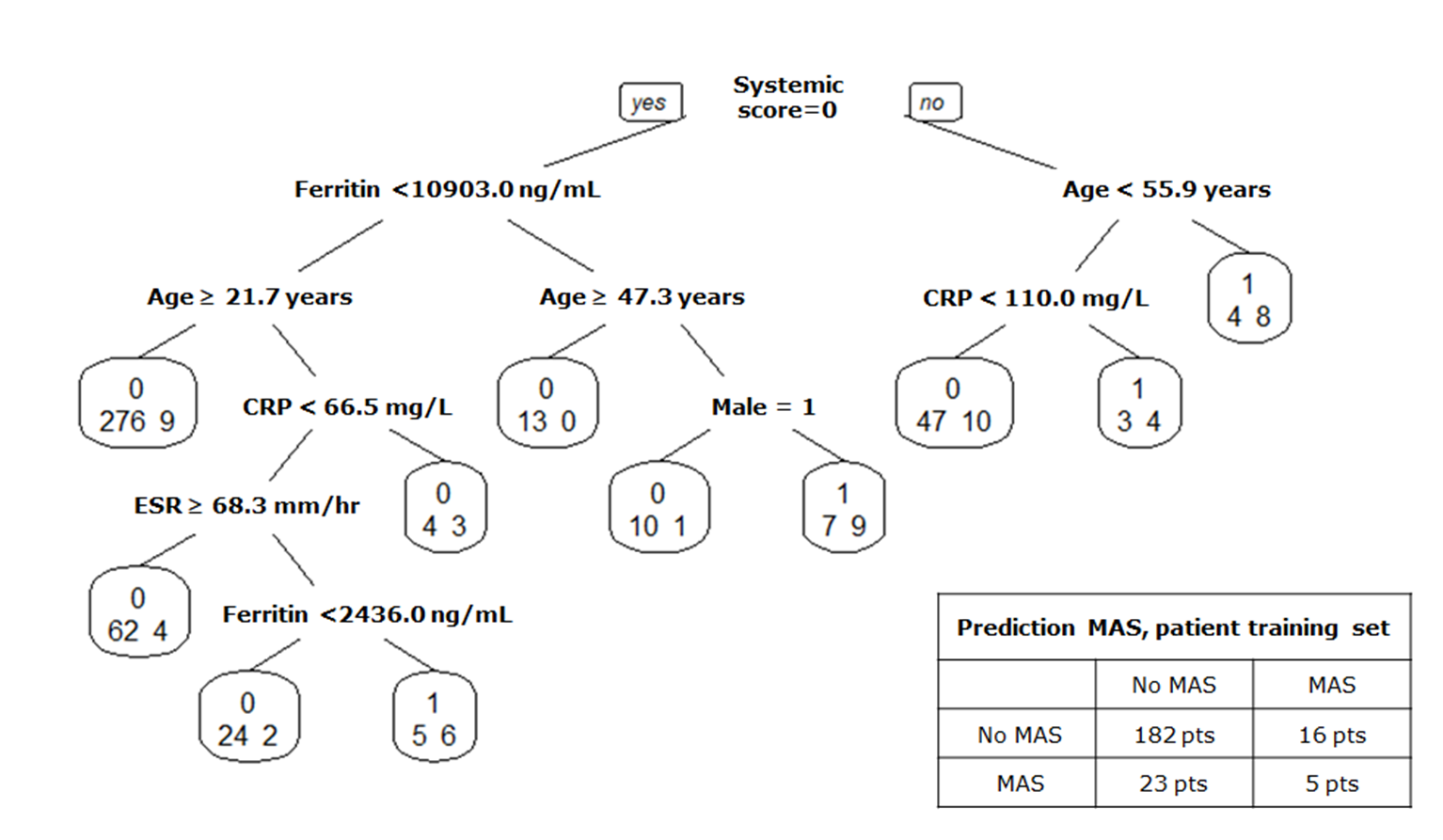

Supplement: Supplementary Figure 1 — An explorative decision tree for the occurrence of MAS. In this figure, the diagnostic role played by patient clinical features has been preliminarily addressed through training a decision tree which, according to the confusion matrix, showed a misclassification error of 17.3%. This assessment suggested the importance of systemic score ≥ 7 in identifying patients with MAS as main node in the decision tree. After that, the role of ferritin and age was highlighted as other important nodes. Consequently, the relevance of age, CRP, and ESR was suggested. In addition, different thresholds for the continuous variables were suggested, thus proposing the diverse significance of the laboratory markers and age according to the clinical scenario. [file Image1.tiff]

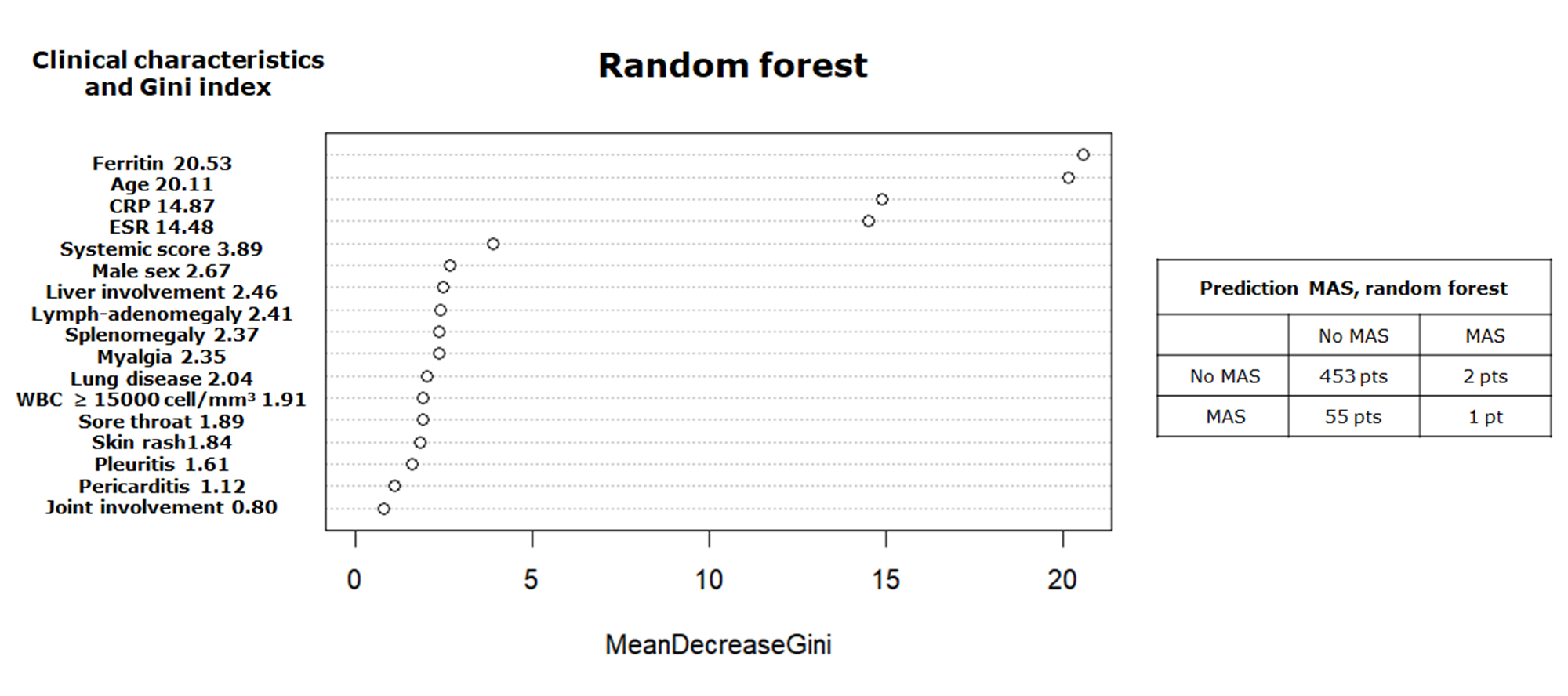

Supplement: Supplementary Figure 2 — Random forest classification performance and Gini’ mean decrease of clinical variables in assessing the risk for the occurrence of MAS. A random forest has been trained on the training set; the random forest classification performance was measured using the OOB (11.1%). The random forest confirmed the relevance of ferritin, age, CRP, ESR, and systemic score. This analysis has also shown the importance of other clinical features in suggesting a more accurate characterization of patients with MAS. The Gini’s mean decrease of assessed clinical variables is also reported in this Figure. [file Image2.tiff]
