# Supplementary material for: Application of machine learning techniques to explore the occurrence of macrophage activation syndrome in Still’s disease: results from the GIRRCS AOSD Study Group and the AIDA Network Still’s Disease Registry
Source: Front Immunol. 2026 Apr 14;17:1811317. doi: 10.3389/fimmu.2026.1811317 (PMC13121312; doi:10.3389/fimmu.2026.1811317)
Supplement: Supplementary file 3 [file Supplementaryfile1.docx]

**Supplementary results 1. Metrics for multivariate model comprising imputed variables to prevent data leakage.**

*ROC area estimation*

ROC Asymptotic normal

Obs area Std. err. [95% conf. interval]

------------------------------------------------------------

510 0.66 0.04 0.58-0.74

Method: Youden

Reference variable: mas (0=neg, 1=pos)

Classification variable: p

Youden index (J): 0.303

SE(J): 0.0668

*Confusion Matrix using Youden Index*

|  | **P score (Youden Index)** | |
| --- | --- | --- |
| **MAS** | 0, No | 1, Yes |
|  |  |  |
| 0, No | 424 | 25 |
| 1, Yes | 45 | 16 |
|  |  |  |

*Performance Metrics*

| **Measure** | **Value** | **95%CI** |
| --- | --- | --- |
| Sensitivity | 0.26 | (0.15–0.37) |
| Specificity | 0.94 | (0.92–0.97) |
| Misclassification error | 0.14 | (0.11-0.17) |
| Positive likelihood ratio (LR⁺) | 4.69 | (2.6–8.4) |
| Negative likelihood ratio (LR⁻) | 0.78 | (0.63–0.97) |

The performance of the test is summarized in this performance metrics table. Sensitivity was 0.26 (95%CI 0.15–0.37), while specificity was high at 0.94 (95%CI 0.92–0.97). The overall misclassification error was 0.14. The positive likelihood ratio was 4.69 (95%CI 2.6–8.4), whereas the negative likelihood ratio was 0.78 (95%CI 0.63–0.97). The estimated parameters showed high specificity but low sensitivity, indicating good ability to correctly identify non-MAS patients but limited capacity to detect cases. The positive likelihood ratio (4.69) indicates that a positive test result increases the post-test probability of MAS occurrence. In contrast, the negative likelihood ratio (0.78) suggests that a negative test result provides limited information for ruling out MAS.
