# Supplementary material for: Application of machine learning techniques to explore the occurrence of macrophage activation syndrome in Still’s disease: results from the GIRRCS AOSD Study Group and the AIDA Network Still’s Disease Registry
Source: Front Immunol. 2026 Apr 14;17:1811317. doi: 10.3389/fimmu.2026.1811317 (PMC13121312; doi:10.3389/fimmu.2026.1811317)
Supplement: Supplementary file 4 [file Supplementaryfile2.docx]

STROBE Statement—checklist of items that should be included in reports of observational studies

|  | Item No. | Recommendation | Page  No. | Relevant text from manuscript |
| --- | --- | --- | --- | --- |
| **Title and abstract** | 1 | (*a*) Indicate the study’s design with a commonly used term in the title or the abstract | 1 | The machine learning driven prediction of macrophage activation syndrome in Still’s disease based on a random forest imputation; results from the GIRRCS AOSD-study group and the AIDA Network Still’s Disease Registry |
|  |  | (*b*) Provide in the abstract an informative and balanced summary of what was done and what was found | 6 | Study abstract |
| Introduction | | | |  |
| Background/rationale | 2 | Explain the scientific background and rationale for the investigation being reported | 8,9 | the application of advanced machine learning models may offer a novel lens to analyse these patients with Still’s disease developing MAS by simultaneously integrating diverse clinical manifestations |
| Objectives | 3 | State specific objectives, including any prespecified hypotheses | 9 | in this work, we aimed to assessing machine learning driven prediction of MAS in Still’s disease based on a random forest imputation in a multicentre, observational, prospective study. We also assessed the survival impact of MAS in this study cohort. |
| Methods9 | | | |  |
| Study design | 4 | Present key elements of study design early in the paper | 9 | A multicenter, observational, prospective study was built up, including patients with Still’s disease who were included in the GIRRCS (*Gruppo Italiano di Ricerca in Reumatologia Clinica e Sperimentale*) AOSD-study group and the AIDA (AutoInflammatory Disease Alliance) Network Still’s Disease Registry. |
| Setting | 5 | Describe the setting, locations, and relevant dates, including periods of recruitment, exposure, follow-up, and data collection | 9,10 | The GIRRCS AOSD-study group cohort is a national Italian multicenter study involving rheumatologic units with experience in management of Still’s disease. Furthermore, patients with Still’s disease were selected from those included in AIDA Network Still’s Disease Registry, an international, clinical, physician-driven, non-population, and electronic-based registry. |
| Participants | 6 | (*a*) *Cohort study*—Give the eligibility criteria, and the sources and methods of selection of participants. Describe methods of follow-up  *Case-control study*—Give the eligibility criteria, and the sources and methods of case ascertainment and control selection. Give the rationale for the choice of cases and controls  *Cross-sectional study*—Give the eligibility criteria, and the sources and methods of selection of participants | 9-11 | Patients were included before the latest EULAR/PReS recommendations and thus children fulfilled specific criteria for sJIA and adults for AOSD, respectively. In all patients, other inflammatory diseases, malignancies, and infections were ruled out as previously detailed. |
|  |  | (*b*) *Cohort study*—For matched studies, give matching criteria and number of exposed and unexposed  *Case-control study*—For matched studies, give matching criteria and the number of controls per case | N/A |  |
| Variables | 7 | Clearly define all outcomes, exposures, predictors, potential confounders, and effect modifiers. Give diagnostic criteria, if applicable | 10,11 |  |
| Data sources/ measurement | 8* | For each variable of interest, give sources of data and details of methods of assessment (measurement). Describe comparability of assessment methods if there is more than one group | *9-11* | In this work, we aimed to assessing machine learning driven prediction of MAS in Still’s disease based on a random forest imputation in a multicentre, observational, prospective study. We also assessed the survival impact of MAS in this study cohort |
| Bias | 9 | Describe any efforts to address potential sources of bias | 11 | During patient scheduled visits, relevant data were collected by reviewing the clinical charts stored in each center. All the data were fully anonymized before the analyses. All data generated by the analysis is included in the body of the present work. |
| Study size | 10 | Explain how the study size was arrived at | 11 | Based on the “real-life” nature of our assessment, no specific sample size was estimated for this study cohort. However, considering the number of assessed patients (n = 737), we had enough power to build up any of our analyses. |

Continued on next page

| Quantitative variables | 11 | Explain how quantitative variables were handled in the analyses. If applicable, describe which groupings were chosen and why |  | N/A |
| --- | --- | --- | --- | --- |
| Statistical methods | 12 | (*a*) Describe all statistical methods, including those used to control for confounding | 11-13 | The statistical analysis was worked out using statistical software R version 4.4.2. The study aimed at selecting and characterizing covariates affecting MAS both in terms of probability of occurrence as well as in terms of survival impact. Both these endpoints faced up to the preliminary issue of missingness. |
|  |  | (*b*) Describe any methods used to examine subgroups and interactions | 11-13 | A random forest model was performed to assess the covariates classification importance, using 500 trees and 4 variables tried at each split. |
|  |  | (*c*) Explain how missing data were addressed | 11-13 | Because of the multivariate distribution to be imputed, to avoid questionable *a priori* distributional hypothesis, we decided to use a nonparametric imputation method which imputed mixed type of variables, accounting for complex interactions, high dimensionality and nonlinear relations among the variables included by the study design. The only reasonable assumption in our setting is that the observations are pairwise independent. OOB imputation error has been estimated (0.18). Stekhoven and Buhlmann gave reasons supporting this estimate as an appropriate representation of the true imputation error. Little’s test for MCAR multivariate distribution of missingness has been preliminarily carried out. We used the R library “missForest” for the imputation and the library “naniar” to perform the MCAR hypothesis test. |
|  |  | (*d*) *Cohort study*—If applicable, explain how loss to follow-up was addressed  *Case-control study*—If applicable, explain how matching of cases and controls was addressed  *Cross-sectional study*—If applicable, describe analytical methods taking account of sampling strategy | N/A |  |
|  |  | (*e*) Describe any sensitivity analyses |  |  |
| Results | | | | |
| Participants | 13* | (a) Report numbers of individuals at each stage of study—eg numbers potentially eligible, examined for eligibility, confirmed eligible, included in the study, completing follow-up, and analysed | 13, Table 1 | In this study, 737 patients (age 35.5 ± 17.8, male sex 44.7%) were assessed, as detailed in Table 1 which reports the clinical characteristics of patients before the imputation. Out of these, 16.7% were pediatrics. |
|  |  | (b) Give reasons for non-participation at each stage | N/A |  |
|  |  | (c) Consider use of a flow diagram | N/A |  |
| Descriptive data | 14* | (a) Give characteristics of study participants (eg demographic, clinical, social) and information on exposures and potential confounders | Table 1 | Table 1 |
|  |  | (b) Indicate number of participants with missing data for each variable of interest | 9-11 | Application of random forest imputation |
|  |  | (c) *Cohort study*—Summarise follow-up time (eg, average and total amount) | Table 1 | Table 1 |
| Outcome data | 15* | *Cohort study*—Report numbers of outcome events or summary measures over time | Table2-5, Figure 1-3 | In this cohort, 11.4% of patients were burdened by MAS and 3% had a poor prognosis; the presence of MAS was estimated to be correlated with mortality (χ^2^=33.3, p<0.001). |
|  |  | *Case-control study—*Report numbers in each exposure category, or summary measures of exposure | N/A |  |
|  |  | *Cross-sectional study—*Report numbers of outcome events or summary measures | N/A |  |
| Main results | 16 | (*a*) Give unadjusted estimates and, if applicable, confounder-adjusted estimates and their precision (eg, 95% confidence interval). Make clear which confounders were adjusted for and why they were included | 13-16  Table2-5, Figure 1-3 | Based on the results of the explorative tree and random forest, the variables age, ferritin, CRP, ESR, and systemic score were identified as main predictors of MAS in our cohort. |
|  |  | (*b*) Report category boundaries when continuous variables were categorized | N/A |  |
|  |  | (*c*) If relevant, consider translating estimates of relative risk into absolute risk for a meaningful time period | N/A |  |

Continued on next page

| Other analyses | 17 | Report other analyses done—eg analyses of subgroups and interactions, and sensitivity analyses | N/A |  |
| --- | --- | --- | --- | --- |
| Discussion | | | | |
| Key results | 18 | Summarise key results with reference to study objectives | 16 | In this study, an accurate machine learning prediction of MAS, based on a random forest imputation, has been provided to increase the accuracy of the detection of this condition, which, despite the recent management improvement, still remains the life-threatening evolution of patients with Still’s disease. Furthermore, age ≥ 45 years, ferritin ≥ 4178.10 ng/ml, CRP ≥ 27.15 mg/L, and systemic score ≥ 7 have been identified as the most relevant clinical characteristics in prediction of MAS and their combinations may delineate some patient subsets with a different risk of this complication in the context of Still’s disease. |
| Limitations | 19 | Discuss limitations of the study, taking into account sources of potential bias or imprecision. Discuss both direction and magnitude of any potential bias | 18 | Our study is affected by different limitations which may reduce the generalizability of the derived results. Despite we assessed a combination of two prospective large cohorts including patients with Still’s disease, the multicenter studies have some inherent limitations regarding the possible differences in clinical practice between centers, in confounding the collection of the data and consequently the interpretation of the results. |
| Interpretation | 20 | Give a cautious overall interpretation of results considering objectives, limitations, multiplicity of analyses, results from similar studies, and other relevant evidence | 18 | in this work, we assessed the machine learning driven prediction of MAS in Still’s disease based on a random forest imputation in a multicentre, observational, prospective study, which highlighted the importance of age of onset, hyperferritinaemia, increased CRP and multi-organ involvement. |
| Generalisability | 21 | Discuss the generalisability (external validity) of the study results | 18 | A combination of these features may suggest a clinician-friendly algorithm in accurately identifying the probability of the presence of MAS during Still’s disease. |
| Other information | |  | | |
| Funding | 22 | Give the source of funding and the role of the funders for the present study and, if applicable, for the original study on which the present article is based | N/A | N/A |

*Give information separately for cases and controls in case-control studies and, if applicable, for exposed and unexposed groups in cohort and cross-sectional studies.

**Note:** An Explanation and Elaboration article discusses each checklist item and gives methodological background and published examples of transparent reporting. Information on the STROBE Initiative is available at www.strobe-statement.org.
